# Livestock embryonic stem cells for reproductive biotechniques and genetic improvement

**DOI:** 10.1590/1984-3143-AR2024-0029

**Published:** 2024-08-05

**Authors:** Micaela Navarro, Lucia Laiz-Quiroga, Carolina Blüguermann, Adrián Mutto

**Affiliations:** 1 Laboratorio de Biotecnologías aplicadas a la Reproducción Animal, Instituto de Investigaciones Biotecnológicas “Dr. Rodolfo Ugalde”, Universidad Nacional de General San Martín, Buenos Aires, Argentina

**Keywords:** embryonic stem cells, genetic engineering, gametogenesis, pluripotency

## Abstract

Embryonic stem cells (ESCs) have proven to be a great *in vitro* model that faithfully recapitulates the events that occur during *in vivo* embryogenesis, making them a unique tool to study the cellular and molecular mechanisms that define tissue specification during embryonic development. Livestock ESCs are particularly attractive and have broad prospects including drug selection and human disease modeling, improvement of reproductive biotechniques and agriculture-related applications such as production of genetically modified animals. While mice and human ESCs have been established many years ago, no significant advances were made in livestock species until recently. Nowadays, livestock ESCs are available from cattle, pigs, sheep, horses and rabbits with different states of pluripotency. In this review, we summarize the current advances on livestock ESCs establishment and maintenance along with their present and future applications.

## Introduction

The first ESCs were derived from the inner cell mass (ICM) of mice embryos (Evans and Kaufman, 1981) and subsequently in rhesus macaque (Thomson et al., 1995) and humans (Thomson et al., 1998). The attempts to derive ESC from livestock began in 1987 (as reviewed by Navarro et al., 2020), a few years after the first derivation of ESC in mice, however ESCs derivation in livestock species remained elusive until recently when genetically stable and bona fide pluripotent stem cells (PSCs) were achieved. While mice and human ESC are widely attractive due to the production of transgenic animals and their use in regenerative medicine, respectively; the appeal of ESC from livestock species lies in their potential use in reproductive biotechniques and genetic engineering. In this review, we summarize the state of art of ESC from livestock species, describing the different pluripotency states that were captured *in vitro*, and demonstrate possible applications in *in vitro* generation of gametes and *in vitro* breeding programs. Finally, we show different approaches for producing genetically modified animals and highlight the advantages of editing ESC instead of somatic cells or embryos.

## Embryonic stem cells in livestock species

Embryonic stem cells can be found at different stages of embryonic development, have an unlimited self-renewal capacity and are pluripotent, meaning that they have the potential to differentiate into every cell type present in an adult organism (Nichols and Smith, 2009). These cells have been classified according to the embryonic state that they represent, being the naïve and the primed states the two most common categories (Nichols and Smith, 2009).

Derivation of ESCs from livestock species has been a long-term challenge due to the elusiveness of culture conditions that maintain the pluripotent state of the cells. So far, five reports of ESCs in cattle (Bogliotti et al., 2018; Kinoshita et al., 2021; Shirasawa et al., 2024; Xiang et al., 2021; Zhao et al., 2021), four in pigs (Choi et al., 2019; Gao et al., 2019; Kinoshita et al., 2021; Zhi et al., 2022), two in sheep (Kinoshita et al., 2021; Vilarino et al., 2020), one in horses (Yu et al., 2021) and one in rabbits (Kobayashi et al., 2021) are available, and even though the conditions under which they were obtained share some aspects, they are different as well as the pluripotent state that they recreate (Table 1).

**Table 1:** Derivation source, cultured conditions and pluripotent state of livestock embryonic stem cells.

**Cell line**	**Species**	**Derivation source**	**Pluripotency state**	**Molecular profile**	**Culture conditions**	**Growth Morphology**	***In vivo* pluripotency assay**	**PGCLC differentiation**	**Components used for PGCLC induction**	**Refences**
CTFR-Bovine Embryonic Stem cells	Bovine	Day 7 blastocyst (*in vitro*) or isolated ICMs	Primed state	OCT4 and SOX2	Custom-made base medium, BSA, FGF2 and IWR1	2D colonies	Teratomas	-	-	Bogliotti et al. (2018)
NBFR-Bovine Embryonic Stem cells		Day 7 blastocysts (*in vitro*)			N2B27, BSA, FGF2 and IWR1				-	Soto et al. (2021)
Bovine Embryonic Stem Cells	Bovine	Isolated ICMs from day 5 embryos (*in vitro*) incubated with C3 and PD0325901	Primed state	OCT4, SOX2, NANOG, CD9, SSEA4 and CD49f/ITGA6	mTeSR1 and IWR1. Once established, media was changed to NBFR	2D colonies	-	Yes	BMP4, SCF, EGF, LIF, CHIR990221 and Y-27632	Shirasawa et al. (2024)
Pig Embryonic Stem Cells	Porcine	Hatched blastocysts (*in vitro*)	Primed state	OCT4, SOX2, NANOG, SSEA1, SSEA4, TRA-1-60, and TRA-1-81	DMEM, KSR, a lipid concentrate, FGF2, ActA, CHIR99021 and IWR1	2D colonies	Teratomas	-	-	Choi et al. (2019)
Expanded Potential Stem Cells (EPSC)	Bovine	Early day 5 blastocyst (*in vivo*)	Expanded potential state	OCT4, SOX2, NANOG, and SALL4	mTESR1, IWR1 (or XAV-939), CHIR99021, WH-4-023 or A419259, Vitamin C, ActA and LIF	3D colonies	Chimeras and teratomas	-	-	Zhao et al. (2021)
	Porcine			OCT4 and NANOG	N2B27, FBS, CHIR99021, WH-4-023, XAV-939 (or IWR1), Vitamin C, ActA and LIF			Yes	BMP2, LIF, SCF and EGF	Gao et al. (2019)
Embryonic Disc Stem Cells (EDSC)	Bovine	Isolated ICMs from day 7-9 blastocysts (*in vitro*)	Primed state	OCT4, SOX2 and NANOG	N2B27, ActA, FGF2 and XAV-939	2D colonies	Teratomas	-	-	Kinoshita et al. (2021)
	Porcine	Epiblasts from bilaminar discs from day 11 embryos (*in vivo*)							-	
	Ovine	Day 7 blastocysts (*in vitro*)							-	
Pre-gastrulation Epiblast Stem Cells (pgEpiSC)	Porcine	Epiblasts from day 10 embryos (*in vivo*)	Formative pluripotency	OCT4, SOX2, NANOG, SSEA1, SSEA4, TRA-1-81, and TRA-1-60	N2B27, KSR, Vitamin C, CHIR99021, IWR1, WH-4-023, LIF, ActA and FGF2	3D colonies	Teratomas	-	-	Zhi et al. (2022)
Sheep Embryonic Stem Cells	Ovine	ICMs from day 7 blastocyst (*in vitro*)	Primed state	OCT4, SOX2 and NANOG	Custom-made base medium, BSA, FGF2 and IWR1	2D colonies	Teratomas	-	-	Vilarino et al. (2020)
Chimera and Primordial Germ Cell Dual-Competent Pluripotent Stem Cells (XPSC)	Equine	Zona-free day 7 blastocyst (*in vitro*)	Formative pluripotency	OCT4 and SOX2	N2B27, FGF2, ActA and CHIR99021	2D colonies	Chimeras and teratomas	Yes	BMP4, LIF, SCF and EGF	Yu et al. (2021)
Rabbit Pluripotent Stem Cells	Rabbit	Day 6 epiblast, isolated day 4 ICMs or whole day 4 blastocysts (*in vivo*)	Primed state	OCT4, SOX2 and NANOG	Essential 8, IWP2 and XAV-939	2D colonies	Teratomas	Yes	B27, BMP4, LIF, SCF and EGF	Kobayashi et al. (2021)

PGCLC: primordial germ cell-like cells; ICM: inner cell mass; BSA: bovine serum albumin; FGF2: fibroblast growth factor 2; IWR1: WNT signaling inhibitor 1; DMEM: Dulbecco′s Modified Eagle′s Medium; KSR: knock-out serum replacement; ActA: activin A; CHIR990221: GSK3 inhibitor; XAV-939: WNT signaling inhibitor; WH-4-023: tyrosine kinases Lck and Src inhibitor; A419259: Src kinases inhibitor; IWP2: WNT signaling inhibitor; LIF: leukemia inhibitory factor; FBS: fetal bovine serum; BMP4: bone morphogenetic protein 4; SCF: stem cell factor; EGF: Epidermal Growth Factor; Y-27632: ROCK inhibitor.

Bovine ESC (bESC) were the first livestock ESCs that accomplished a true and stable pluripotent state *in vitro*. They were derived in 2018 by Bogliotti et al. (2018) using CTFR culture medium, which is a custom-made base medium (similar to mTeSR1) supplemented with low fatty acid bovine serum albumin (BSA), fibroblast growth factor 2 (FGF2) and the inhibitor of the Wnt/Tankyrase pathway IWR1, and the use of mitotically inactivated mouse embryonic fibroblasts (iMEF) as feeder layers. For isolating bESCs, three approaches were tested: plating the whole day 7 *in vitro* produced blastocyst or plating ICMs that were isolated by immunosurgery or mechanically; although the efficiency of the procedure did not change between methods. Bovine ESCs formed 2D colonies, had a normal karyotype and showed a high proliferation kinetic along multiple passages (>50). Regarding their pluripotent state, they showed positive staining for alkaline phosphatase and for the pluripotency markers SOX2 and OCT4, and when injected into immunodeficient mice, they formed teratomas with tissues from the three germ layers, although no chimerism capability was tested. However, since their transcriptomic and epigenomic landscape revealed that they resembled the primed state, it would be likely that these cells do not harbor chimerism capacity.

A few years later, the same group reported a simplification of the culture conditions used for deriving and culturing bESC (Soto et al., 2021). Instead of using the CTFR medium and iMEF as feeder layer, a chemically defined media (called NBFR) and recombinant human vitronectin were proposed. NBFR medium consisted of N2B27 media supplemented with low fatty acid BSA, FGF2 and IWR1. These conditions have been proven to efficiently derive bESC, with no differences between using whole day 7 *in vitro* produced blastocysts or isolated ICMs. The bESC lines had similar features compared to CTFR-bESC derived cells: they showed expression of pluripotency markers, had a normal karyotype, exhibited a similar epigenetic pattern that was related to the primed state, and formed teratomas containing derivatives from the three germ layers. Afterwards, this group aimed to replace the iMEF with a defined matrix. They demonstrated that these cells could be adapted to feeder-free conditions without losing their pluripotency hallmarks. Notably, it was necessary to include Activin A (ActA) in the culture media formulation when using feeder-free conditions, indicating that this factor probably plays a critical role in the maintenance of bovine pluripotency. This work was pioneer in simplifying bESCs culture conditions and in demonstrating that bovine pluripotency and self-renewal was dependent on FGF2, ActA and Wnt/Tankyrase pathways. Some years later, the same group demonstrated that these simplified conditions were also useful for deriving bESCs from different embryonic sources, including *in vitro* fertilization (IVF), parthenogenesis and somatic cell nuclear transfer (SCNT) (Navarro et al., 2022).

In the same year of bESC culture simplification, another type of bESCs that had the ability of contributing to embryonic and extraembryonic lineages was described by Zhao et al. (2021). This type of cells, named expanded potential stem cells (EPSC), had previously been isolated in mice and humans (Yang et al., 2017a, b), and subsequently in pigs (Gao et al., 2019). Bovine EPSCs were derived by plating *in vivo* embryos at the early blastocyst stage from different breeds, on mTESR1 medium supplemented with IWR1 (or XAV-939, which is another Wnt/Tankyrase inhibitor), CHIR99021 (glycogen synthase kinase 3β inhibitor), WH-4-023 or A419259 (dual Lck/Src inhibitors), Vitamin C, ActA and leukemia inhibitory factor (LIF) on irradiated bovine fetal fibroblasts. These cells formed compact domed colonies and had transcriptomic and epigenetic features similar to early-stage preimplantation embryos. They could be cultured long-term without altering their karyotype, they expressed pluripotency genes and could be adapted to feeder-free conditions. Upon *in vivo* differentiation, these cells were able to contribute to embryonic and extraembryonic tissues in chimeras, although with low efficiency. Finally, these cells could be genetically edited and used as donors during the nuclear transfer (NT) technique, where the resulting embryos could be used again for deriving new bovine EPSCs.

Also in 2021, another group derived a new type of bovine stem cells but different from the others previously described (Kinoshita et al., 2021). These cells called embryonic disc stem cells (EDSC) transcriptionally resembled the bilaminar disc epiblast and were derived from isolated ICMs from day 7-9 blastocysts in culture conditions that were similar to the previously described NBFR conditions, although IWR1 and vitronectin were replaced for XAV-939 and a mix of fibronectin/laminin matrix, respectively. Interestingly, these culture conditions were also suitable to derive EDSC from sheep and pig. Authors demonstrated that derivation efficiency varied according to the embryonic stage, being E8 and E9 bovine blastocysts the most suitable stages for achieving a higher efficiency. Similar to other reports in cattle, bovine EDSCs exhibited pluripotency features, were stable for over 30 passages, and were able to differentiate into the three germ layers *in vitro* and *in vivo*. Finally, authors demonstrated that bovine EDSCs could be differentiated into skeletal muscle lineage, being a starting point for their use in cellular agriculture.

Recently, Shirasawa et al. (2024) established new bESCs lines which shared some characteristics with the bESC described by Soto et al. (2021), but with some differences at the initial stages of derivation (Shirasawa et al., 2024). During this work, authors identified that the incubation of day 5 embryos with C3 (inhibitor of rho-associated protein kinase (ROCK) by C3 transferase) and PD0325901 (inhibitor of FGF pathway) for 4 days increased the number of epiblasts cells compared to trophectoderm cells. These ‘epiblast-improved’ embryos were used for deriving bESC, where the presence of C3 inhibitor also improved the derivation efficiency without altering their transcriptome. For ESCs derivation, immunosurgery isolated ICMs were plated on iMEF feeders in mTeSR1 medium supplemented with IWR1. Once established, media was changed to NBFR media, as in Soto et al. (2021). Similar to other reports in cattle, these cells showed similarities with the primed state and expressed pluripotency markers. Interestingly, these cells were differentiated to primordial germ cell-like cells (PGCLCs), a fact that was not previously demonstrated in cattle and that paves the way for the use of bESC on reproductive biotechnologies.

In addition to cattle, successful attempts were made on pigs as well. In 2019, the first two reports of stable pig ESCs (pESCs) were published. In one report, pESC derivation was performed by plating hatched blastocysts on iMEF in knock-out Dulbecco’s Modified Eagle’s Medium (DMEM) supplemented with knock-out serum replacement (KSR), a lipid concentrate, FGF2, ActA, CHIR99021 and IWR1 (Choi et al., 2019). On the second report, early blastocysts were plated on a mouse feeder layer, with N2B27 medium supplemented with fetal bovine serum, CHIR99021, WH-4-023, XAV-939 (or IWR1), Vitamin C, ActA and LIF (Gao et al., 2019). In both cases, cells expressed pluripotency markers, had a normal karyotype, produced teratomas with derivatives of the three germ layers and were *in vitro* cultivated for multiple passages. Even though both pESCs showed pluripotency features, the ones derived by Choi et al. (2019) were closer to the primed state, whereas the ones derived by Gao et al. (2019) represented an earlier stage since they had the capability to also contribute to extraembryonic lineages when injected into chimeras.

Two years later, the group of Kinoshita et al. (2021), who described EDSCs in cattle, also achieved this state in pigs by using the same culture conditions (Kinoshita et al., 2021). In this case, bilaminar discs from *in vivo* day 11 embryos were manually dissected, and the epiblasts were plated to derive pEDSCs. Similar to cattle, these cells showed pluripotency features and genome stability. Particularly, these cells were successfully genetically edited and used as donors for NT.

The latest report of pESC was in 2022 by Zhi et al. (2022), who described the pre-gastrulation epiblast stem cells (pgEpiSC), cells that transcriptionally resembled pre-gastrulation pig embryos and held formative pluripotency. To obtain these cells, authors mechanically isolated epiblasts from day 10 *in vivo* embryos and plated them on iMEF with N2B27 medium supplemented with KSR, Vitamin C, CHIR99021, IWR1, WH-4-023, LIF, ActA and FGF2. These cells were stable for over 240 passages while showing pluripotency hallmarks. While their chimerism capacity was not achieved, these cells could be used as donors for NT, even after multiple rounds of gene editing.

While these advances have been made in cattle and pig, few reports are available for other livestock species. In sheep, there are two studies that succeeded in obtaining ESCs (sESCs). The first report of sESCs was in 2020 by the same group that first reported bESCs (Vilarino et al., 2020). In the same study, sheep ICMs were plated on iMEF with CTFR medium and after 3 weeks, sESCs formed dome-shaped colonies which expressed pluripotency markers and had a normal karyotype even after long-term culture. These cells had a transcriptome compatible with the primed state, and when injected into immunodeficient mice, they formed teratomas with tissues from the three germ layers. Similar to cattle, these cells were successfully adapted to vitronectin-coated plates only when culture media was supplemented with ActA. One year later, the same group that described EDSCs in cattle and pigs also obtained these cells in sheep (Kinoshita et al., 2021). The same culture conditions were used for the three species and the resulting cells showed the same characteristics. Noteworthy, it was only possible to obtain sheep EDSC from embryos at E7, whereas no colonies were observed when using E6 embryos. Particularly, these cells seemed more likely to differentiate compared to other species, which made it necessary to carefully isolate the undifferentiated cells to prevent differentiated cells from dominating the culture.

In 2021, a novel method was described for obtaining ESC in the formative state in humans, mice and horses (Yu et al., 2021). Stable equine ESCs, known as chimera and primordial germ cell dual-competent PSCs, were obtained by plating zona-free blastocyst on iMEF or Matrigel with FTW media which consisted of N2B27 medium supplemented with FGF2, ActA and CHIR99021. Cells accomplished pluripotency hallmarks and had a transcriptomic pattern related to the formative state, a state between naïve and primed pluripotency states. These cells were able to not only incorporate into intra- and inter-species chimeras, but also to differentiate to PGCLCs under bone morphogenetic protein 4 (BMP4) stimulation.

PSCs that have shown their potential to produce PGCLCs were also achieved in rabbits (Kobayashi et al., 2021). Epiblast of E6 embryos, isolated ICMs or whole blastocysts at day 4 were cultured on mice feeders in Essential 8 medium supplemented with two Wnt inhibitors: IWP2 and XAV-939. Cells had a normal karyotype, expressed pluripotency markers and could be differentiated *in vitro* and *in vivo*. Their transcriptomic information revealed that these cells had an expression profile similar to that of a rabbit disc-shaped *in vivo* epiblast.

After the first report of ESCs in cattle, many other reports have been published in livestock. While each report has isolated ESCs that represent different stages of embryo development, most of them are related to the primed state, and it seems necessary to modulate the FGF, Wnt/Tankyrase and ActA pathways to maintain their self-renewal and pluripotency state *in vitro*. The availability of ESCs in livestock species will allow for a better understanding of the main pathways involved in embryo development and could enable the use of *in vitro* breeding strategies, *in vitro* gametogenesis and genetic improvement.

## Embryonic stem cells for reproductive biotechniques in livestock

The main goal of livestock breeding is to improve genetic merit by selectively mating animals with desirable traits to enhance future generations' performance and productivity. Thanks to the development of high-throughput genotyping techniques, modern breeding programs reduce generational intervals by selecting based on genetic markers (Hu et al., 2016). However, these programs continue to have a slow development due to the inherently long gestational periods and the time required to reach puberty. Thus, it seems logical to look for new technologies aiming to accelerate breeding times.

**Figure 1:**
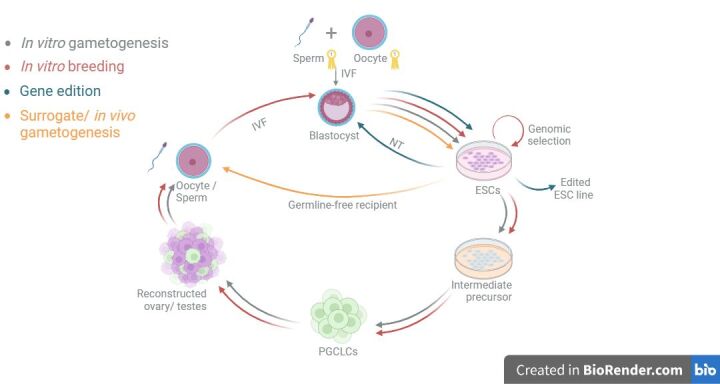
Germ cell specification pathway and the different applications of each step. ESCs could be obtained from in vitro produced embryos by IVF from elite individuals. ESCs could be used for in vitro breeding and be subjected to different rounds of genomic selection (arrows in red), and/or for gene editing (arrows in blue). These cells could be further used as nuclear donors in NT, or used for producing surrogate parents (arrows in orange), or they could continue through a differentiation pathway towards germ cell specification (arrows in gray). Following germ cell specification, these cells could produce gametes in vitro to generate new embryos, and start the cycle again. Figure created with BioRender (2024).

After the successful derivation of ESCs in livestock species, new methods such as ‘In Vitro Breeding’ have been proposed to leverage this advancement and improve animal breeding programs (Goszczynski et al., 2019). This method is based on deriving ESCs from embryos with a high genetic value, where each cell line would be genotyped to select the best genetic combinations. These selected ESCs lines would then be differentiated *in vitro* into oocytes and sperms, where these gametes would be used for IVF to produce a new round of embryos (Figure 1). It is estimated that embryo production through IVF and ESCs derivation would take around 4 weeks in cattle plus 3 months to obtain mature gametes, meaning that each generation would take about 3 or 4 months, considerably less time than traditional breeding (~2.5 years). Although this approach is revolutionary for animal agriculture, there is still a lot of work to be done, mainly related to the development of *in vitro* gametogenesis procedures.

*In vitro* gametogenesis (IVG) consists in producing functional gametes *in vitro* from different sources of stem cells, being the PSCs the ones with the best results to date. *In vitro* gametogenesis in livestock not only has the potential to improve genetics through selective breeding but also enables endangered species conservation, supports agricultural advancements by preserving unique livestock breeds, and aids in biomedical research through the creation of disease models.

A few years ago, the entire cycle of the mouse germ line was reconstituted *in vitro* for the first time (Hikabe et al., 2016; Li et al., 2019). Mouse ESCs were differentiated in two steps to obtain functional gametes able to produce healthy offspring. The first step consisted in differentiating naïve ESCs to an advanced developmental stage called epiblast-like cells (EpiLC). The next step was to differentiate EpiLCs to PGCLCs, the precursors of gametes, under BMP2/4 proteins. PGCLCs were then co-cultivated with somatic cells from fetal ovaries or testis to induce the differentiation, growth, and maturation of the haploid gametes. With these encouraging results in rodents, many attempts were made for reproducing these protocols in humans, achieving great advances although recapitulation of gametogenesis has not been fully achieved *in vitro* yet (Hwang et al., 2020; Yamashiro et al., 2018). In livestock, akin to the situation observed with ESC derivation, IVG has not advanced to such an extent.

The first report that aimed to produce PGCLCs was carried out in pigs (Gao et al., 2019). In this study, authors made a NANOS3 reporter EPSC line (which is a conserved PGC marker) to identify the induction of the germ cell lineage. By doing a single or combined transient expression of SOX17, BLIMP1, NANOG, and/or TFAP2C, they observed an increased number of NANOS3 positive cells when SOX17 and BLIMP1 were present, opposite to what was reported in humans where the expression of BLIMP1, NANOG and TFAP2C induced expression of NANOS3. However, similarly to other species, the response to BMP proteins has been shown to be conserved in pigs. After cultivating the reporter lines in the presence of BMP2, LIF, stem cell factor (SCF) and epidermal growth factor (EGF), the expression of PGC markers was detected, including OCT4, NANOG, LIN28A, TFAP2C, CD38, DND1, NANOS3, ITGB3, SOX15 and KIT, together with a reduction of SOX2 expression. Nevertheless, the induction efficiency was relatively low. Recently, Pieri et al. (2022) attained swine PGCLCs that had an expression pattern similar to *in vivo* isolated PGCs. In this case, authors used induced pluripotent stem-like cells to produce PGCLCs and culture conditions similar to those used to obtain mice PGCLCs.

Encouraging results were also published in horses (Yu et al., 2021) and rabbits (Kobayashi et al., 2021) where acquisition of primordial germ cell potential was induced directly through BMP stimulation, without the necessity of going through an intermediate epiblast-like state. Specifically in rabbits, going through an intermediate state decreased the germ cell induction efficiency (Kobayashi et al., 2021). In horses, reporter ESCs lines at the formative state were cultured in GK15 media supplemented with BMP4, LIF, SCF and EGF. Authors observed that after one day of treatment, cells expressed PGCs markers such as SOX17, TFAP2C, PRDM1, NANOS3, KIT, DND1, UTF1, MSX2 and DPPA3, being the expression of these markers even higher after 2-3 days (Yu et al., 2021). Interestingly, the expression profile of PGCLCs from mice, humans and horses revealed that the last ones were more similar to human germ cells compared to mice, suggesting that similar pathways could be conserved across both species. In the case of rabbits, for PGCLCs induction, reporter ESCs lines were cultured in the presence of DMEM/F12 medium supplemented with B27 supplement, BMP4, LIF, SCF and EGF (Kobayashi et al., 2021). The resulting cells expressed PGC markers like TFAP2C, PRDM1, OCT4, NANOG, SOX17, KLF4 and TFCP2L1, and did not express SOX2. Transcriptomic analysis showed that rabbit PGCLCs were equivalent to *in vivo* PGC at a peri-gastrulation stage, indicating a successful differentiation of the cells.

Until recently, there were no advancements towards the recapitulation of the germ cell cycle in cattle. However, in 2024, Shirasawa et al. (2024) reported the *in vitro* production of bovine PGCLCs from primed ESCs. In this case, a two-gene reporter line (BLIMP1 and TFAP2C) was created to identify PGCLCs. For PGCLC induction, contrary to rabbits and horses, authors made a pre-incubation step that consisted on culturing ESCs on a fibronectin-coated plate in the presence of N2B27 medium supplemented with KSR, BMP4, CHIR99021, IWR1 and a ROCK inhibitor. Twenty-four hours later, cells were cultured in GK15 with BMP4, SCF, EGF, LIF, CHIR99021 and IWR1 to induce germ cell specification. Interestingly, a single stimulation with BMP4 was not enough to induce germ cell differentiation. However, if BMP4 stimulation was accompanied with a Wnt/Tankyrase antagonist and agonist at the same time, germ cell lineage induction was observed, although it was necessary to adjust the concentration of each factor to induce an efficient differentiation of each ESC line. PGCLCs had a transcriptome similar to other mammalian species except from mice, where upregulation of PRDM1/BLIMP1, SOX17, TFAP2C, NANOS3 and KIT was observed, together with downregulation of SOX2. Finally, considering the low availability of antibodies to isolate PGCLCs in most livestock species, authors identified the PGCLCs population as a KIT positive and CD44 negative cell type, which represents a useful tool for future studies.

Overall, it seems that regardless of the pluripotent state that livestock ESCs have, most of them respond to BMP stimulation. Particularly in cattle, the simultaneous stimulation and inhibition of the Wnt/Tankyrase pathway seem to trigger germ cell specification. Future studies should be conducted to understand the mechanisms behind PGC specification and to increase the induction efficiency, which is still relatively low and varies between cell lines significantly.

Considering that IVG in livestock is still not a fully understood process with a lot of research pending to be made, other options to produce gametes of elite animals have been proposed. Different approaches combined with gene editing sought the use of germ cells-deleted embryos for hosting the production of germ cells of a desired animal. This strategy has been used before for regenerative medicine, where wild type ESCs were used for complementing an embryo lacking *pdx1*, the gene responsible for pancreas generation. In this work, it was demonstrated that when ESCs were injected into a *pdx1^-/-^* blastocyst, they mostly contribute to the pancreas formation even when the ESCs were from a related but different species (Kobayashi et al., 2010). Taking this background into consideration, many reports focused on the depletion of the germ cell lineage without affecting the further physiology of the tissue. NANOS gene plays a vital role in germ cell development in several species. In mammals, NANOS2 and NANOS3 homologs have been identified to be specifically expressed in germ cells (Tsuda et al., 2003). Therefore, many attempts have been made to eliminate NANOS3 or NANOS2 to obtain germ-cell ablated individuals. In pigs, sheep and cattle it was demonstrated that a NANOS2 knockout resulted in a phenotype similar to mice NANOS2 deficient. In males, the homozygous knockout derived in germ cell ablation but in morphologically normal seminiferous tubules, while heterozygous male and female as well as homozygous females were fertile (Ciccarelli et al., 2020; Mueller et al., 2023; Park et al., 2017). On the other hand, NANOS3 knockout was done in pig and cattle where the resulting individuals had a similar phenotype to mice: there were no germ cells while the gonadal development remained unaltered (Ideta et al., 2016; Kogasaka et al., 2022; Mueller et al., 2023; Park et al., 2022; Wang et al., 2023). Having accomplished a completely germ cell free individuals, the next step would be to complement these embryos with ESCs, and to investigate whether these embryos could host donor-derived exogenous germ cells, generating the opportunity to produce gametes from genetically desirable individuals. While this strategy seems promising, it has to be considered that ESC chimera contribution could be the limiting step for its further application.

While there is still much work to be done to achieve functional gametes in most mammalian species, we believe that it is only a matter of time until this procedure could be finally achieved. In the meantime, there are alternatives to IVG that can be applied to *in vitro* breeding programs to continue with the cycle and still result in a faster genomic dissemination. As mentioned before, one option would be using surrogate parents for producing the gametes of interest. Another option would be using the genomic selected ESCs lines as donors for NT, a fact that has been demonstrated before in livestock (Kinoshita et al., 2021; Zhao et al., 2021; Zhi et al., 2022). The last one could also be combined with the genomic edition of the ESCs lines to assure an even faster genomic improvement (Figure 1).

## Embryonic stem cells for livestock genetic improvement

Since the first report of transgenic mice (Gordon et al., 1980), several animals have been modified with different purposes. In particular, advancements in genetic engineering of large animals were primarily motivated by biomedical purposes rather than agricultural research. These purposes included producing pharmaceutical proteins in the milk of ruminants, modeling human diseases, producing organs for xenotransplantation, among others (Niemann and Kues, 2007). Later on, generation of edited animals pursued the modification of economically important traits such as growth rate (Hammer et al., 1985), resistance to diseases (Wall et al., 2005), increasing meat quality (Saeki et al., 2004), or to facilitate animal handling (Carlson et al., 2016).

The first genetically engineered large animal was produced using microinjection techniques (Hammer et al., 1985). Hammer and collaborators microinjected DNA of the fusion gene MT–hGH13 (metallothionein-I promoter/regulator region fused to human growth hormone) into the zygote pronuclei or nuclei of eggs from super-ovulated sheep, rabbits and pigs. As a result, they obtained transgenic animals able to express the human protein. Since then, the journey has been challenging due to the limited genetic tools available. The development of efficient pronuclear microinjection, combined with the availability of ESCs and omics data, allowed rapid progress in mice gene editing, however, similar progress has been lacking, to some extent, in the realm of livestock.

The feasibility of creating desired animal models depended on the available technologies, which often had low efficiencies. The initial milestone was reached through the advancement of SCNT, which allowed for the selection of genetically modified clones (Wilmut et al., 2015). SCNT paved the way for creating livestock species with precise genetic alterations, such as gene knockouts (Fischer et al., 2020), conditional targeting (Li et al., 2015), and gene insertions (Rieblinger et al., 2018). Despite the benefits of SCNT, generating fully edited embryos for the trait of interest remained a difficult task since SCNT embryos usually show signs of developmental defects related to inefficient reprogramming of the somatic cell used as nuclear donor, regardless of whether the donor cell was wild type or genetically modified (Wilmut et al., 2015). The challenges faced in large animal genetic engineering have been overcome by the availability of stem cell lines and novel genome editing technologies. The development of more efficient editing techniques such as Clustered Regularly Interspaced Short Palindromic Repeats- associated protein 9 (CRISPR/Cas9) technology has revolutionized livestock genetic edition by offering unparalleled efficiency through RNA-based DNA recognition coupled with Cas nucleases, allowing for multiple mutations (Fischer et al., 2020). Its user-friendly design and cost-effectiveness have made it the preferred system for genetic engineering. Furthermore, the emergence of CRISPR/Cas9 technology has potentially rendered previous methods, such as zinc-finger nucleases or transcription activator-like effector nucleases (TALENS), that became obsolete due to their complexity and high cost.

**Figure 2:**
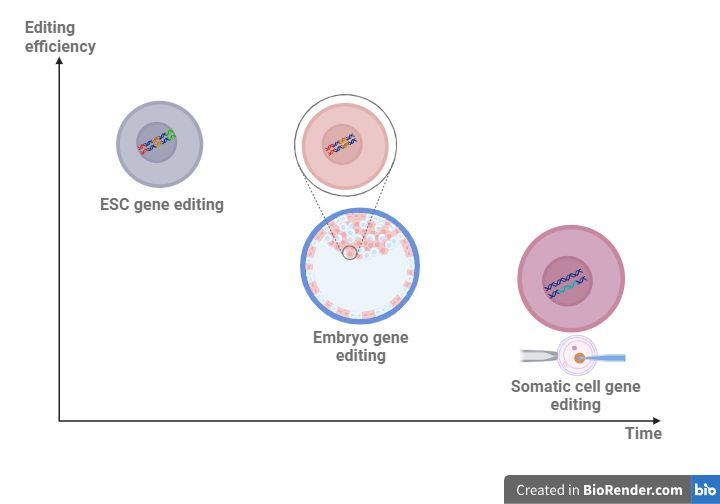
Gene editing using different approaches. The use of ESCs as a tool for gene editing has advantages in terms of efficiency and time of generating a desired individual, compared to other cell types like embryo gene editing or somatic cell editing. ESCs can be edited multiple times (shown in different colors in the DNA) and these cells could be used later as donors in NT. On the other hand, embryo editing often presents a high prevalence of mosaicism (shown as cells in different colors) with lower acceptance for multiple editions. Finally, somatic cell editing allows single editions due to the short lifespan of these cells, and then these cells could be used as donors in SCNT. Figure created with BioRender (2024).

Even with the advent of CRISPR/Cas9, creating animals with multiple genetic modifications by SCNT are both time-consuming, inefficient and resource-intensive (Figure 2). When using somatic cells, one would have to first edit the cells, select and isolate the bi-allelic edited clone, use these edited cells as nuclear donors in SCNT, wait for the generated animal to reach puberty and then mate to introduce and disseminate a desired genetic in the flock. Although this approach is useful for a single-gene edition, performing multiple editions in primary cultures is very challenging due to its limited lifespan, requiring several rounds of SCNT and cell rejuvenation.

Another alternative sought in editing the embryos directly, either at zygote stage or after the first cleavage. Traditionally, embryo editing is done by microinjecting early stage embryos (zygotes or 2/4 cells stage embryos) which requires sophisticated equipment and highly trained personnel. However, simpler methods such as electroporation are now available aiming to replace traditional techniques, showing a great success in livestock (Betters et al., 2018; Camargo et al., 2020; Mahdi et al., 2022). Although this editing methodology would be useful for introducing several mutations, it is prone to result in a high proportion of mosaic embryos, resulting in low efficiency outcomes, even when using CRISPR/Cas9 (Figure 2). It has been reported that Cas9 protein exhibits a delayed activity, being able to produce modifications at first stages of development, resulting in a high degree of mosaicism. To partially overcome this difficulty, CRISPR/Cas9 introduction was performed at matured oocytes, achieving more encouraging results (Vilarino et al., 2017).

The derivation of ESCs from different livestock species has been proposed as a new tool to overcome these difficulties (Figure 2). In particular because of its unlimited replication capacity, the use of ESCs would allow multiple genetic modifications, something very challenging to obtain using primary cultures or embryos. ESCs could be derived from high genetic embryos and edited several times while maintaining genomic stability. Thereafter, edited ESCs could be used as nuclear donors for NT or in IVG (Figure 2). The fact that ESCs can be used as donors for NT was already demonstrated, although it remains to be determined whether their use results in a higher efficiency in embryo production than using somatic cells (Bogliotti et al., 2018; Kinoshita et al., 2021; Zhao et al., 2021; Zhi et al., 2022).

The capability of ESCs for supporting gene editing was first demonstrated in mice (Templeton et al., 1997). Recently and together with the attainment of livestock ESCs, many attempts have been made on these species. In most reports in livestock, the edition of ESCs was used as a strategy to identify PGCLC precursors (Gao et al., 2019; Kinoshita et al., 2021; Shirasawa et al., 2024; Yu et al., 2021; Zhao et al., 2021). A recent work in pigs, showed that pgEpiSC readily tolerated at least three rounds of successive gene editing modifications, including traditional transgenic insertion, precision knock-in with CRISPR/Cas9 and single-base conversion editing using cytosine base-editors. Similar to other reports in livestock, the edited cells, when used for NT, were able to generate cloned gene-edited live piglets, proving that edited ESC could be successfully used to generate complex pig models (Zhi et al., 2022).

In all, these findings demonstrated that the edition of ESCs is an excellent tool for biological research, animal husbandry, and regenerative biomedicine. The possibility of using ESCs in combination with modern editing techniques and whole genome sequence opens the door to considering performing gene editing of complex traits that could be associated with more than one single mutation.

## Conclusions

The establishment of *in vitro* culture conditions for the maintenance of bovine embryonic stem cell lines paved the way for the establishment of stem cell lines in different livestock species. Since then, different pluripotency states were captured *in vitro* and ESCs from horses, pigs, sheep and rabbits became available. These cell lines not only facilitate the research of embryonic development in livestock species, but also provide new resources for the generation of novel biotechnological tools such the production of gametes *in vitro* and genetically edited animals. As described in the present work, culture conditions to establish livestock ESCs have in common the regulation of the FGF, TGFβ and Wnt/Tankyrases signaling pathways as a requirement for the maintenance of the transcriptional program of the pluripotent state. Despite four decades of effort, robust propagation of pluripotent stem cells from livestock remains challenging and much research is still needed, especially in some species such as sheep, horses and goats for which there are few or no reports on the derivation of these cells.

## Future perspectives

The field of application of ESCs is expanding and has broadened the horizons of biotechnology. If stable cultures of naïve pluripotent stem cells could be established for livestock species (i.e. with the capacity to contribute to chimeras), these cells could be used as germ cell donors when injected in germ cells-depleted embryos. Additionally, the recapitulation of the whole male and female germ cell cycle *in vitro* would allow their use in *in vitro* breeding programs, which in combination with genome editing, could enable the modification of complex characteristics in livestock animals and strongly improve genetic value in considerably less time. The development of cutting-edge single-cell multi-omics technologies would facilitate the discovery of new mechanisms involved in livestock germ cell specification, which is crucial for going a step further in the process of *in vitro* gametogenesis.
